# Characterizing Infections in Two Epidemic Waves of SARS-CoV-2 Omicron Variants: A Cohort Study in Guangzhou, China

**DOI:** 10.3390/v16040649

**Published:** 2024-04-22

**Authors:** Lin Qu, Chunyan Xie, Ming Qiu, Lina Yi, Zhe Liu, Lirong Zou, Pei Hu, Huimin Jiang, Huimin Lian, Mingda Yang, Haiyi Yang, Huiling Zeng, Huimin Chen, Jianguo Zhao, Jianpeng Xiao, Jianfeng He, Ying Yang, Liang Chen, Baisheng Li, Jiufeng Sun, Jing Lu

**Affiliations:** 1 School of Public Health, Sun Yat-sen University, Guangzhou 510080, China; quinnyl@163.com (L.Q.); churdeming@163.com (M.Q.); yanghy99@mail2.sysu.edu.cn (H.Y.); 2 Guangdong Provincial Institution of Public Health, Guangzhou 511430, China; 18720795430@163.com (C.X.); linayi2009@live.cn (L.Y.); lzhwin@foxmail.com (Z.L.); wmkong1005@163.com (H.J.); lianlhm@163.com (H.L.); 19867687957@163.com (M.Y.); 13163764733@163.com (H.Z.); c13694991170@163.com (H.C.); zhao_jg95@163.com (J.Z.); jpengx@163.com (J.X.); yang99063@126.com (Y.Y.); 18928929722@126.com (L.C.); 3 Guangdong Workstation for Emerging Infectious Disease Control and Prevention, Guangdong Provincial Key Laboratory of Pathogen Detection for Emerging Infectious Disease Response, Guangdong Provincial Center for Disease Control and Prevention, Guangzhou 511430, China; zou_lirong@hotmail.com (L.Z.); hjf@vip.sina.com (J.H.); libsn@126.com (B.L.); 4 School of Basic Medicine and Public Health, Jinan University, Guangzhou 510632, China; 5 Guangdong Provincial Center for Disease Control and Prevention, Guangzhou 511430, China; hupei8866@163.com; 6 School of Public Health, Southern Medical University, Guangzhou 510515, China; 7 School of Public Health, Guangdong Pharmaceutical University, Guangzhou 510310, China

**Keywords:** SARS-CoV-2, Omicron variant, cohort study, viral trajectories, neutralizing antibodies

## Abstract

Background: After the adjustment of COVID-19 epidemic policy, mainland China experienced two consecutive waves of Omicron variants within a seven-month period. In Guangzhou city, as one of the most populous regions, the viral infection characteristics, molecular epidemiology, and the dynamic of population immunity are still elusive. Methods: We launched a prospective cohort study in the Guangdong Provincial CDC from December 2022 to July 2023. Fifty participants who received the same vaccination regimen and had no previous infection were recruited. Results: 90% of individuals were infected with Omicron BA.5* variants within three weeks in the first wave. Thirteen cases (28.26%) experienced infection with XBB.1* variants, occurring from 14 weeks to 21 weeks after the first wave. BA.5* infections exhibited higher viral loads in nasopharyngeal sites compared to oropharyngeal sites. Compared to BA.5* infections, the XBB.1* infections had significantly milder clinical symptoms, lower viral loads, and shorter durations of virus positivity. The infection with the BA.5* variant elicited varying levels of neutralizing antibodies against XBB.1* among different individuals, even with similar levels of BA.5* antibodies. The level of neutralizing antibodies specific to XBB.1* determined the risk of reinfection. Conclusions: The rapid large-scale infections of the Omicron variants have quickly established herd immunity among the population in mainland China. In the future of the COVID-19 epidemic, a lower infection rate but a longer duration can be expected. Given the large population size and ongoing diversified herd immunity, it remains crucial to closely monitor the molecular epidemiology of SARS-CoV-2 for the emergence of new variants of concern in this region. Additionally, the timely evaluation of the immune status across different age groups is essential for informing future vaccination strategies and intervention policies.

## 1. Introduction

Since late 2019, SARS-CoV-2 has caused 771 million infections and 6.9 million fatalities [1]. The virus has undergone rapid evolution and positive selection with worrisome variants (Alpha, Beta, Gamma, Delta, and Omicron) emerging and exhibiting increased fitness, transmissibility, and immune evasion [2]. Omicron has successfully displaced Delta and rapidly become the dominant strain worldwide [3]. The Omicron variant has given rise to several sublineages, including BA.2, BA.4, BA.5, BA.7, BF.1, and XBB.1, with some sublineages being more prevalent in certain areas than others. 

In mainland China, strict non-pharmaceutical interventions (NPIs)—dynamic zero-COVID prevention and control policy—had been implemented [4] since the end of 2019. Most localized COVID-19 outbreaks were primarily attributed to imported cases and the scale of transmission remained relatively limited [5]. On 7 December 2022, most of the NPIs, including social distancing, mass testing, and quarantine, were lifted [6] in China due to the high vaccination rate (a total of 34.88 billion doses of COVID-19 vaccines were administered by January 2023 [7]) and the decreased viral pathogenicity [8]. Thereafter, two Omicron variants—BA.5 (mainly circulating in Southern China) and BF.7 (mainly circulating in Northern China)—have been responsible for a sharp rise in infections in early 2023, and the XBB.1 variants epidemic in the middle of 2023. 

The ongoing pandemic of SARS-CoV-2 has not been halted, and diverse population immunity backgrounds can result in varying viral infection trajectories and the dynamic evolution of the virus. To date, there have been limited longitudinal studies conducted on the situation following the surge of the BA.5 and XBB.1 epidemic in mainland China. As a result, crucial aspects such as viral infection dynamics, clinical implications, and the herd immune response in this highly populated region remain largely unknown. 

On 10 December 2022, three days after the discontinuation of the dynamic zero-COVID policy, we initiated a cohort study in the Guangdong Provincial Center for Disease Control and Prevention (GDCDC). The study included institutional staff who had no previous history of SARS-CoV-2 infection and had received three doses of inactivated vaccine by the end of October 2022. The Guangdong Provincial Vaccination System reports that 79.07% of the population were administered three doses of the inactivated vaccine from October 2021 to October 2022. As such, the immune profile of the individuals within the cohort likely mirrors that of the broader population within the same age brackets. In this research, we outlined the distinct features of infection symptoms and the progression of viral infection kinetics among the cohort participants who endured two rounds of the Omicron epidemic. Moreover, we evaluated the neutralization antibody titers of these individuals following the two epidemic waves. This was done to examine the ways in which SARS-CoV-2 variant infections shaped individual immunity and to explore their potential interactions. 

## 2. Materials and Methods

### 2.1. Study Design and Participants

**Figure 1:**
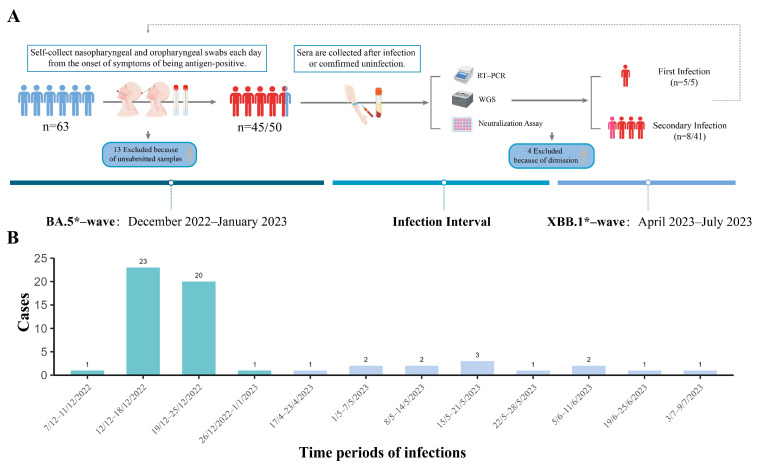
Overview of the study cohort. (**A**) Nasopharyngeal/oropharyngeal swabs were collected when suspected symptoms appeared or when the antigen was positive in each period and were sent to the laboratory within 24 h for RT-PCR and WGS analysis to obtain Ct values and genetic sequences of SARS-CoV-2. Convalescent blood samples were collected from all patients (28 days after the Ct value turned negative) and healthy people (uninfected as determined by antigen tests), and sera were isolated for live authentic viruses microneutralization tests to detect the specificity of the patients against different variants of SARS-CoV-2. Participants who did not submit swab samples at enrollment (*n* = 13) were excluded from all analyses, and 4 individuals were excluded from the second analysis due to dimission before the second epidemic. (**B**) The confirmed infections in this cohort study from 7 December 2022 to 9 July 2023. The green column represents the first wave (caused by BA.5* variants), and the blue column is the second (caused by XBB.1* variants). RT-PCR, reverse transcription polymerase chain reaction; WGS, Whole Genome Sequencing; Ct, cycle threshold. The asterisk represents the lineage of SARS-CoV-2 and its subvariants.

All participants underwent almost daily nucleic acid testing during the COVID-19 pandemic. The testing system recorded that none of them had a history of SARS-CoV-2 infection and had received three doses of inactivated vaccine (CoronaVac, Sinovac Life Sciences Co., Ltd., Beijing, China) by the end of October 2022. After the lifting of the strict NPIs, the population has experienced two epidemic waves dominated by the different representative Omicron variants circulating from December 2022 to July 2023. The first period is referred to as the BA.5*-wave, and the second is known as the XBB.1*-wave (the asterisk represents the lineage of SARS-CoV-2 and its subvariants) (Figure 1). All participants had no history of mental illness, cancer, immune deficiency disease, or use of immunosuppressants or glucocorticoids in the last 3 months. Each participant was trained to self-collect nasopharyngeal and oropharyngeal swabs, and self-monitoring was performed by reporting the daily antigen test results and symptoms in an online spreadsheet, which included demographic information and a pre-defined set of symptoms (fever, cough, asthenia, headache, anosmia or taste abnormality, and so on). For individuals detected to be SARS-CoV-2 antigen-positive or who developed any related clinical symptoms, nasopharyngeal and oropharyngeal swabs were collected during the infection and were transported to the laboratory of the GDCDC in a cold chain for RT-PCR and WGS analysis within 24 h. In addition, convalescent blood samples were collected from all infected cases at 28 days after the Ct (Cycle threshold) value turns negative. For XBB.1*-uninfected individuals whose antigen tests were negative during the second epidemic, serums were collected at the end of the second epidemic wave on 21/25 July.

### 2.2. RT-PCR

Nasopharyngeal and oropharyngeal swab samples underwent nucleic acid extraction using the TIANLONG Viral RNA extraction kit (CDC) (PANA9600E) on the TIANLONG GeneRotex 96 Automatic nucleic acid extractor following the instrument’s recommendations. The real-time reverse transcription PCR (RT-PCR) was performed using the commercial kit (20203400212, Wuhan Easy Diagnosis Biomedicine Co., Ltd., Wuhan, China) and a RT-PCR machine (CFX96, Bio-Rad Laboratories, Hercules, CA, USA) in the same method as the previous studies [9]. For quality control, each time the clinical samples were tested, the aliquoted positive and negative controls of the kit were performed through RNA extraction and RT-PCR along with the samples. The positive control was a constructed pseudovirus including the RT-PCR targeting fragments. As in the previous study [9], the viral load quantification was performed through the serially diluted the reference pseudovirus (Reference Material no. GBW(E)091132, Guangzhou BDS Biological Technology Co., Ltd., Guangzhou, China), of which digital RT-qPCR determined the absolute copies of N.

### 2.3. Whole Genome Sequencing, WGS

Whole-genomic sequencing was performed using the Nanopore Rapid Barcoding Sequencing (SQK-RBK004) protocol on a GridlON X5 (Oxford Nanopore, Oxford, UK) sequencing instrument as previously described [10]. Briefly, cDNA was generated from the previously extracted RNA remaining after the initial diagnostic RT-PCR assay. The hexamer cDNA was randomly synthesized using SuperScript IV (Thermo Fisher, Waltham, MA, USA, 18091) for two-step reverse transcriptase PCR. For Oxford Nanopore sequencing, amplicon pools were indexed using the Rapid Sequencing Adapter (Oxford Nanopore, Oxford, UK, EXP-RAP001). Then, about 400 ng of the resulting library was used for sequencing on Oxford Nanopore GridION instruments using R9.4.1 flow cells. Clade determination was performed via NextClade CLI v2.14.1 [11].

### 2.4. Phylogenetic Analysis

For the phylogeny, 58 complete or near-complete SARS-CoV-2 genome sequences from 50 cohort individuals were generated and combined with reference sequences of major lineages (according to the reference sequences in the nextclade dataset, https://clades.nextstrain.org/, accessed on 10 November 2023). The phylogenetic tree reconstruction was performed using NEXTSTRAIN’s (https://www.nextstrain.org/, accessed on 10 November 2023) augur (https://docs.nextstrain.org/projects/augur/en/stable/, accessed on 10 November 2023) pipeline. Multiple alignment was performed by using Mafft and using MN908947 as the reference. Maximum-likelihood (ML) trees were estimated by using FasttreeMP and refined with Treetime to infer the molecular-clock phylogeny. Finally, the collection of all annotated nodes and metadata was exported to the interactive phylodynamic visualizing tool Auspice’s (https://auspice.us/, accessed on 10 November 2023) in a JSON format.

### 2.5. Microneutralization Detection

The SARS-CoV-2 neutralization test was conducted in the Biosafety Level III Laboratory of the Guangdong Center for Disease Control and Prevention (BSL-3), and the neutralization was determined according to the literature [12]. With Omicron sublineages (BA.5, GDPCC 2.00303; XBB.1.9, GDPCC 2.01543) as the target virus, serum samples were continuously diluted 2 times with the minimum necessary medium, from concentrations of 1:4 to 1:1024. The diluted serum was mixed with 100 SARS-CoV-2 TCID50 and incubated at 37 °C for 1 h. The mixture was added to VeroE6 cells and incubated at 37 °C and 5% carbon dioxide. After incubation for 7 days, the cytopathic effect was detected under an inverted microscope. The titer dilution of the neutralization antibody (nAb) was the highest, and the inhibition rate of the cytopathic effect was 50%.

### 2.6. Statistical Analysis

Data are reported as means ± standard deviation or medians (IQRs) for continuous parameters and frequency (%) for categorical variables. χ^2^ and Fisher’s exact tests were used to compare categorical variables; for continuous variables, the *t* test was used for normal data and the Mann–Whitney U-test was used for non-normal data. The paired Wilcoxon signed-rank test method was used to compare neutralization titers (convalescent sera from two waves for the same person). The trajectory of change in viral Ct values at symptom onset is characterized by using a generalized additive model (GAM) and a log link with a smoothing spline placed on time (measured in days) to allow for non-linear effects. This allows for overall and individual viral trajectories to be modeled. All analyses were done using R version 4.2.2 and GraphPad Prism version 9.5.1 for Windows. A two-sided *p*-value of less than 0.05 was considered significant.

## 3. Results

### 3.1. Epidemiology Characters

As presented in Figure 1, the initial recruitment of our cohort study comprised 63 participants (23 male and 40 female participants), all of whom received three doses of inactivated vaccines by the end of October 2022. Thirteen participants in the first wave and four participants in the second wave were excluded due to unsubmitted specimens. A total of 45 COVID-19 infections were identified in 50 cohort individuals during the first wave, and 13 in 46 cohort individuals were detected as positive during the second wave in which 8 cases were reinfected (Figure 1). 

**Figure 2:**
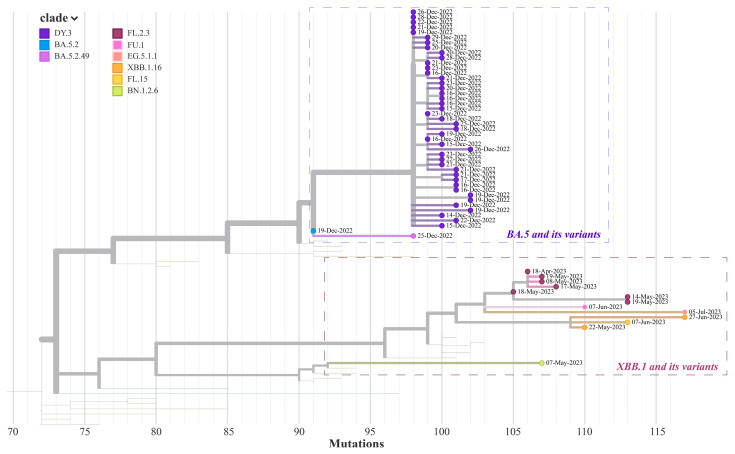
Viral phylogenies of two Guangzhou Omicron outbreaks. A time-resolved phylogenetic tree was estimated using the NextStrain pipeline and includes all infection sequences (*n* = 58) collected from our Guangzhou cohort, December 2022–July 2023. Each point represents the sequence of an infected individual, and the distance between the point and the root node indicates the number of nucleotide mutations between it and the root sequence (MN908947). The SARS-CoV-2 lineages are classified according to the pangolin classification scheme, https://github.com/cov-lineages/pangolind, accessed on 21 September 2023.

The genetic sequencing showed most individuals (43 out of 45, 95.56%) in our cohort were infected with DY.3 variants, a sublineage of BA.5.2.48, and two individuals were infected with BA.5.2 and BA.5.2.49 which were divergent from the major circulating variants of BA.5.2.48 in the first wave. Consistent with the sharp increasing of the epidemics, viral genomes from DY.3 (representing the major clade as mention above)-infected cases were closely related with a maximum of 4 substitutions that were detected in 43 infections. In contrast, the viral genetic lineages collected during the second wave were more diversified in spite of there being fewer infection cases. Six SARS-CoV-2 variants including FL.2.3, FL.15, EG.5.1.1, XBB.1.16, FU.1, and BN.1.2.6 were detected in 13 infections during the second wave (Figure 2).

In the BA.5*-wave, the most frequent symptoms were fever (97.78%), asthenia (86.67%), and sore throat (75.56%), cough (73.33%), rhinorrhea (68.89%) and myalgia (68.89%). Compared to the Omicron BA.5* wave, XBB.1* infections in the second wave were limited and sustained for a long period of time. The XBB.1* infection cases presented more different and milder symptoms, characterized by sore throat (69.23%), and cough (61.54%) (*p* > 0.05), and the proportion of patients with a fever (53.85%) was lower (*p* < 0.001). In addition, symptoms of headache and rash were observed in the second wave (*p* < 0.01). The average number of days having self-collected nasopharyngeal/oropharyngeal swabs was 6.69 ± 1.28 during the first wave and 5.92 ± 2.06 in the second (Table 1).

### 3.2. Viral Trajectories of Different Variant Infections in Various Sample Types

**Figure 3:**
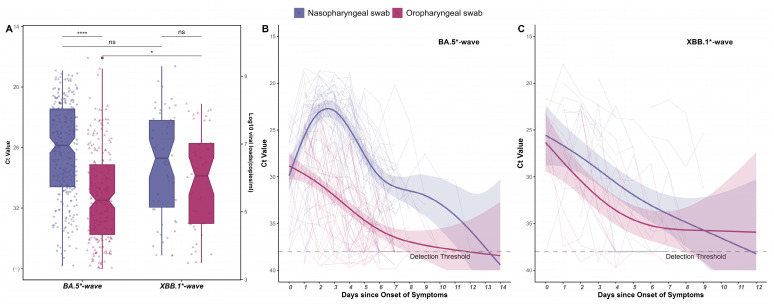
Quantitative results of nasopharyngeal swabs and oropharyngeal swabs of infections in the two waves. (**A**)Two-tailed Wilcoxon signed-rank test is used to compare Ct values for positive nasopharyngeal and oropharyngeal swabs and shows significant changes in Ct values of the same swabs in different waves. Dots represent each Ct value for the RT-PCR of the N gene. Box plots indicate the median, 25th and 75th percentiles, and minimum and maximum values. The vertical axis on the right-hand side gives the conversion from Ct values to RNA concentration. ns, not significant. *, *p* < 0.05. ****, *p* < 0.0001. (**B**,**C**) Temporal profile of serial Ct values from infected individuals. Longitudinal PCR testing was used for cohort individuals (*n* = 45 people in the first wave, 13 in the second), overall and stratified by the swabbing method. The detection threshold was Ct = 38. The thick lines with 95% CIs shown as shading show the trend in viral load, using a generalized additive model (GAM) for smoothing. The different color lines represent the changes over time in the nasopharyngeal and oropharyngeal swabs of cases in the BA.5*-wave (**B**) and XBB.1*-wave (**C**).

The trajectory of viral loads provides insights into the dynamics of virus infectivity and transmission risks. In detail, we collected a total of 281 nasopharyngeal samples (267 PCR+ test) and 298 oropharyngeal samples (213 PCR+ test) in the first wave, 76 nasopharyngeal samples (54 PCR+ test) and 77 oropharyngeal samples (46 PCR+ test) in the second, respectively (Table 1). By comparing positive Ct values of nasopharyngeal and oropharyngeal swabs, we found that in the first wave for BA.5* infections, the viral loads in the nasopharyngeal swabs (Ct = 25.78 for N gene, equivalent to ~9.1 × 10^6^ copies/mL, *IQR* 22.17–29.89) were 41 times higher than those in the oropharyngeal ones (Ct = 31.23 for N gene, equivalent to ~2.2 × 10^5^ copies/mL, *IQR* 27.70–34.63), and the difference between the two was statistically significant (*p* < 0.0001). No significant difference (*p* > 0.05) was observed between the nasopharyngeal (Ct = 27.07, equivalent to ~3.8 × 10^6^ copies/mL, *IQR* 23.31,31.92) and oropharyngeal swabs (Ct = 28.79, equivalent to ~1.2 × 10^6^ copies/mL, *IQR* 25.58,33.52) for XBB.1* infections, possibly due to the limited sample size (*n* = 13). We also compared the viral loads for different variants of infections, and only oropharyngeal swabs showed a statistically significant difference (*p* < 0.05) (Figure 3A). 

To further determine the trend of viral loads over time, we connected the daily nasopharyngeal swabs and oropharyngeal swabs of each individual separately, taking the day of symptom onset as day 0, and constructing a generalized additive model (GAM) to predict the nose and oropharyngeal swab changes as a whole change in viral load. In the BA.5*-wave, it was estimated that the viral RNA peaked at the day of symptom onset (Ct = 28.83, 95%CI: 27.41–30.27) for oropharyngeal specimens. On the other hand, the viral loads in nasopharyngeal sites reach their peak in 2.48 days (Ct = 22.73, 95%CI: 21.84–23.61) after the occurrence of symptoms, highlighting the difference of viral load trajectories in these two respiratory sites (Figure 3B). Furthermore, the viral shedding intervals, from the onset of symptom to the first PCR- test, had an estimated duration of 13.29 days and 11.87 days, for nasopharyngeal and oropharyngeal swabs, respectively (Figure 3B), suggesting a more durable replication and transmissibility for BA.5 and its variants in the human nasopharyngeal cavity. In contrast, in the XBB.1*-wave, the viral loads of nasopharyngeal and oropharyngeal swabs showed a downward trend from the onset of symptoms (Ct = 25.56, 95%CI: 22.24–28.77 vs. Ct = 26.31, 95%CI: 23.25–29.42) (Figure 3C). Notably, the viral shedding interval for nasopharyngeal swabs was approximately 11.70 days, shorter than that in the BA.5*-wave.

### 3.3. Changes in Convalescent Sera-Neutralizing Antibodies in the Cohort Population after the Two Epidemics

**Figure 4:**
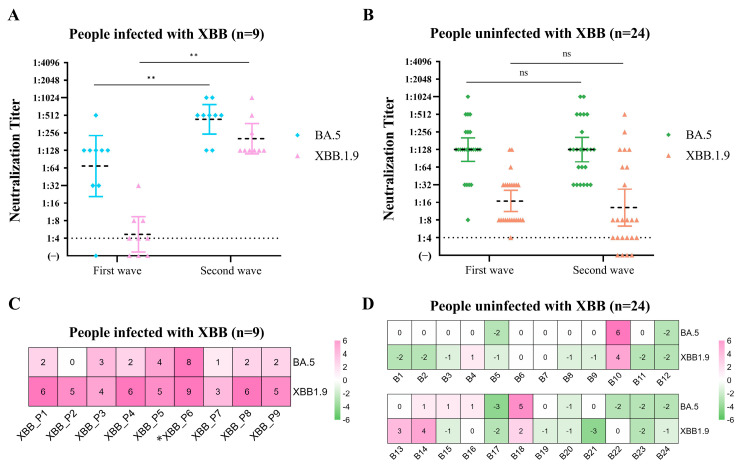
Neutralizing antibody titers and their changes against BA.5 and XBB.1.9 in two waves. (**A**,**C**) Individuals that were infected with XBB (*n* = 9). The asterisk indicates the individual who was not infected in the BA.5*-wave. (**B**,**D**) Individuals that were not infected during the XBB.1*-wave of COVID-19 (*n* = 24). (**A**,**B**) Plots depict the individual neutralizing antibody titers against two variants displayed as neutralization titers. Paired Wilcoxon signed-rank test is used to compare nAbs for the same variants in two waves. Dots of different colors represent convalescent sera nAb titers at two different stages. Error bars represent geometric mean titer (GMT) and its 95% confidence interval. The detection threshold was titer = 1:4. ns, not significant. **, *p* < 0.01. (**C**,**D**) Heatmaps show the degree of change in nAb titers against the same variant in sera after two epidemics. Values in boxes are the log2-transformed ratio of the nAb titers to two waves of XBB.1.9 and BA.5 (e.g., the figure is marked as 1, the actual titer changes by 2 times; marked as 2, by 4 times, and so forth.). >0 (pink background) means an increase in titers, <0 (green background), a decrease, and = 0 (white background), no change.

After analyzing the clinical features and viral load dynamics of the two epidemics in our cohort, we observed that compared with the BA.5* infections, the XBB.1* infections resulted in milder clinical symptoms, lower viral loads, and shorter durations of virus shedding. To explore the interactions between SARS-CoV-2 variant infections and humoral immunity, we further assessed the neutralization antibody titers to BA.5 and XBB.1.9 post the first and the second epidemic waves in 33 individuals. For all the convalescent sera in response to BA.5* infections (*n* = 32), the geometric mean titer (GMT) of the naturalizing antibody (nAb) was 108.2 after the first epidemic wave. As expected, the nAb titer of these sera responses to XBB.1.9 was significantly lower (GMT = 11.92) due to the immune evasion of XBB.1* variants. Although, in the lower nAb titer to XBB.1* following the BA.5* infections, in the XBB.1* epidemic, only 9 XBB infections were identified between April and July in our cohort, comprising eight reinfections and one initial infection of XBB.1*. Twenty-four (24/33, 72.73%) individuals were XBB.1* null infected throughout the study period (Figure 4A–D). All individuals recruited in this cohort study are staff in Guangdong CDC. There were no strict measures to wear masks in public places, and everyone worked and ate in the same building. We suspected each individual in our cohort had an equal chance of being exposed, and the exposure levels were relatively similar among the individuals. To understand the potential relationship between the nAb titers and the susceptibility of the following infections, we compared the nAb titers of BA.5 and XBB.1.9 between the sera collected from XBB-infected and -uninfected individuals at the time before the second wave. Notably, the nAb titers to XBB.1.9 for individuals infected with XBB.1* (GMT = 4.67) in the following epidemic were significantly lower than the individuals who were null infected (GMT = 16.95) (*p* < 0.05). In contrast, the nAb levels to BA.5 for these two groups were similar (*p* > 0.05) (Figure 4A,B). Our results highlighted that the cross-reactive nAbs, representing the breadth of immune response, raised by BA.5* infections may vary among individuals in spite of the same vaccination history. And, the level of cross-reactive nAbs against variant strains determines an individual’s susceptibility to future outbreaks of the virus.

How reinfection of XBB.1* structured individual immunity was still elusive. The vast majority of nAb titers against XBB.1.9 were near or below the limit of detection post the first epidemic (GMT = 4.67, 95%CI: 2.24–9.74), then showed a significant advantage (*p* < 0.01) and exhibited a 43.51-fold increase after XBB.1* infection (GMT = 203.19, 95%CI: 108.02–382.20) (Figure 4A Pink dots). In addition, the XBB.1* infections also raised the nAb titers (GMT = 438.91, 95%CI: 236.67–813.96) against BA.5, with an ~6.35-fold increase compared to the titers of serum collected before XBB.1* infections (GMT = 69.12, 95%CI: 19.26–248.02), (*p* < 0.01) (Figure 4A Blue dots). The reinfection of XBB.1* would have the nAb level against XBB.1.9 (Median = 32, 95%CI: 16–64) rising much more than that of BA.5 (Median = 4, 95%CI: 2–16) (*p* < 0.01, Mann–Whitney U-test) (Figure 4C). Interestingly, P6, who was initially infected with XBB, showed much higher titers against both BA.5 and XBB.1.9 than other reinfected individuals (Figure 4C). 

In the XBB-uninfected group, the nAb titers to BA.5 were comparable for the serums collected after the first and the second epidemic waves (GMT = 128.00, 95%CI: 81.07–202.10 vs. GMT = 128.00, 95%CI: 79.16–207.00) (Figure 4B Green dots), suggesting the persistence of nAb to BA.5 following the BA.5* infections. Over Three-fifths of individuals (*n* = 15) experienced an at least 2-fold decrease in XBB.1.9 nAb titers, among which the decrease mostly remained intact or decreased in the BA.5 nAb titers. Notably, for four individuals, especially B10/13/14/18, not only did their levels of nAb to the XBB.1.9 change more than quadruple, but they also remained or increased against BA.5 (Figure 4D), which shows similar conditions to those of the XBB-infected group. Therefore, we reckon that these four individuals may have been infected with XBB or its subvariants, but were asymptomatic or with viral loads that were undetectable.

## 4. Discussion

It has been over three years since the COVID-19 pandemic was first identified in December 2019 [13]. Due to the implementation of the dynamic zero-COVID policy and the vigorous promotion of the three-shot inactivated vaccine in China, the country experienced its first nationwide wave of COVID-19 caused by the Omicron variant considerably later than other nations [14]. After the release of “the 10-point measures” in China at the end of 2022 [6], the Omicron variant spread widely and simultaneously among the general population, causing two epidemic waves [15] during the observation period of our cohort from December 2022 to July 2023. Aligned with previous modeling assumptions [16], the BA.5* infection rate in our cohort population reached 90% in three weeks following the lifting of NPIs. Possibly influenced by previous BA.5 infection, the reported infection rate (28.26%) during the XBB.1*-wave three months after the peak of the first epidemic wave was significantly lower than that of BA.5 infections. In addition, the clinical symptoms, viral dynamics, and neutralization antibodies of cohort participants were systematically analyzed between these two waves of Omicron infections.

For clinical symptoms, real-world evidence on the disease severity of Omicron variants is scarce. In our study, we found that compared with infections of BA.5*, XBB.1* infections had milder symptoms, lower viral loads, and a short period until turning negative. The BA.5* wave occurred within approximately 3 weeks of the policy change, indicating a relatively high transmission rate of Omicron BA.5* and that boost vaccination provides very limited protection from BA.5* infection due to the long interval from the last dose of vaccination to infection (was at a median of 13.77 months) and the immune evasion of BA.5*. During the second wave, 28.26% (13 in 46) infections were detected, and the two most obvious upper respiratory symptoms, sore throat and cough, were not statistically significant compared with those of BA.5*. The proportion of fever and weakness symptoms in XBB.1* infections was significantly reduced suggesting that milder symptoms resulted from XBB.1* infections.

For viral load trajectories, a few of studies have been performed on BA.5 lineages or XBB.1 lineages. In other regions or countries, the individuals may have diverse immunity backgrounds due to their different infection or vaccination histories which may confound viral load measurements [17]. In our cohort study, all individuals were uninfected with SARS-CoV-2 at the time of enrollment and all completed their three-dose vaccination by the end of October 2022 similar to the majority of the population in mainland China. Our results showed that for Omicron infections, regardless of the variants, the viral load in the nose was significantly higher than that in the oropharyngeal after symptom onset. This is consistent with the replication fitness of Omicron observed in a nasopharyngeal organoids infection model [18,19]. By analyzing the replication dynamics during the entire infection process, we were able to obtain a complete and clear picture of the viral replication kinetics for Omicron variants in the host. For BA.5* infections, the peak viral load was reached in about 2.5 days and viral clearance took about 13 days, with clearance taking 12 days in the oropharyngeal site. Limited by the sampling before symptom onset, it is still uncertain what the viral loads were in oropharyngeal and nasopharyngeal sites before symptom onset. However, it can be preliminarily speculated that the viral load in oropharyngeal specimens may have reached its peak before symptoms appeared, as multiple studies have shown that Omicron is highest 2–5 days after symptoms [20,21] and declines significantly after around 10 days [22], with the virus being detected early in the oropharynx, but at higher and more persistent levels in the nose [23]. Similarly, for XBB.1*cases, we observed that the clearance period of nasopharyngeal swabs was 11.87 days, which was shorter than that of BA.5* infections. The lower viral loads in both oropharyngeal and nasopharyngeal sites and the shorter clearance period may have contributed to the relatively low infection rate of XBB.1* in the second epidemic wave. This replication advantage of Omicron in the nasopharyngeal cavity supports the use of existing intranasal vaccines that exploit mucosal immunity to mediate the body’s response [24]. Existing in vivo experiments show that nasopharyngeal spray vaccines can quickly induce the body to clear the virus and produce long-lasting and broad immunity [25]. Now, there are more than 100 nasopharyngeal spray vaccines under development [26], and 16 are in the clinical trial stage, among which the vaccines from Bharat Biotech and Beijing Wantai Biotech have been approved for emergency use [27]. These could be the tools of choice for counteracting the Omicron epidemic in the near future.

The genetic analysis of cohort samples indicated that the founder effect was the primary driver of the first epidemic wave. The genetic diversity of causative viruses was very limited, mainly consisting of BA.5 and its sublineages with 1–2 substitutions. However, the second epidemic wave was mainly led by XBB.1* variants, which are the dominant variants circulating globally. We are concerned about how the individual’s immunity was structured during these two epidemic waves and the factors related to the susceptibility to reinfections. Consistent with other studies, the XBB.1* variants such as XBB.1 and XBB.1.5 exhibit a strong evasion of the nAbs raised by the wild-type strains or Omicron BA.5 variants [28]. Interestingly, the nAbs analysis of individuals only infected with XBB.1* shows that individuals who have experienced XBB.1* infections elicit strong cross-neutralizing reactions not only to XBB.1.9 but also to BA.5 strains. The wide immunity elicited by XBB.1* infections provides supporting evidence for the updated monovalent COVID-19 mRNA vaccines in 2023–2024, which include XBB.1.5 in the formula [29]. By comparing the nAb titers in the XBB.1*-infected group and the uninfected group before the XBB.1* infection, the lower XBB.1.9 nAb titer (GMT = 4.67) in the infected group compared to the 16.95 in the uninfected group is one possible reason why they got infected in the following epidemic wave. In our cohort, we observed that four XBB.1*-null-infected individuals exhibited neutralization titers against BA.5* and XBB.1* that increased at a similar rate to those seen in XBB.1*-infected cases. This suggests that these individuals may have had asymptomatic infections, highlighting the milder symptoms associated with XBB.1* infections in the current population of mainland China.

Two waves of the epidemic swept across the nation from December 2022 to July 2023, and according to our cohort results, it appears that around 90% of the individuals have been infected with BA.5* and at least 28% of individuals have been infected with XBB.1*. Herd immunity has been achieved through the administration of three doses of inactivated virus vaccines and widespread infections of the two SARS-CoV-2 variants. In future outbreaks, we anticipate a relatively lower infection rate for other variants, such as the EG.5 variant or recently emerged variants, as the epidemic wave flattens. Despite the lower infection rate, the large population size and the ongoing diversified herd immunity may lead to the emergence of new adaptive variants in this region. 

Currently, COVID-19 no longer constitutes a public health emergency of international concern (PHEIC) [30]. The strict COVID-19 control measures have been lifted and the public panic about COVID-19 has greatly reduced in mainland China. However, it remains crucial to closely monitor the molecular epidemiology of SARS-CoV-2 to assess vaccination decisions and the effectiveness of deployed vaccines in a timely manner. Additionally, the continuous immunological monitoring of human sera from different age groups can provide valuable insights into the immune status of the general population [31], serving as a strong foundation for current epidemic prevention and control policies.

### Limitation of the Study

Some limitations of our study should be acknowledged. First, the participants in our cohort were all public health workers aged between 23 and 53. Therefore, the infection characteristics and immune responses observed in this study may not be generalizable to the entire population. It is important to further investigate the status of more vulnerable age groups, such as individuals over 60 years old. Second, in our microneutralization assay, we did not test all the SARS-CoV-2 variants identified in the cohort study. Instead, we selected the representative strains with the highest proportions for the experiment. However, we believe that the nAb titers to BA.5 and XBB.1.9 should be representative of other BA.5* and XBB.1* variants, respectively, since no significant antigenic differences were identified among variants in these two lineages [32]. Finally, due to the limited sample size (*n* = 50), this study observed fewer infected patients during the XBB.1* epidemic, which may, to some extent, underestimate the true infection situation.

## Figures and Tables

**Table 1:** Demographic and clinical data for individuals with COVID-19 infection in two waves.

Characteristics	BA.5*-Wave (*n* = 45) No. (%)	XBB.1*-Wave (*n* = 13) No. (%)	*p* Value
Age, Mean ± standard deviation	32.51 ± 8.54	29.00 ± 5.22	
Sex			
M	16 (42.22)	5 (38.46)	
F	29 (64.44)	8 (61.54)	
Variant			
Omicron BA.5 variants			
BA.5.2	1 (2.22)	0 (0.00)	
BA.5.2.49	1(2.22)	0 (0.00)	
DY.3	43 (95.56)	0 (0.00)	
Omicron XBB.1 variants			
FL.2.3	0 (0.00)	7 (53.85.69)	
FU.1	0 (0.00)	1 (7.69)	
EG.5.1.1	0 (0.00)	1 (7.69)	
XBB.1.16	0 (0.00)	2 (15.38)	
FL.15	0 (0.00)	1 (7.69)	
BN.1.2.6	0 (0.00)	1 (7.69)	
Months between infection and the last dose of vaccination, Median (IQR)	13.77 (11.92,14.02)	17.53 (15.32,19.22)	
Symptoms when diagnosed with COVID-19			
Fever (>37.3 °C)	44 (97.78)	7 (53.85)	<0.001
Cough	33 (73.33)	8 (61.54)	>0.05
Asthenia	39 (86.67)	3 (23.08)	<0.001
Anosmia or taste abnormality	10 (22.22)	4 (30.77)	>0.05
Nasal obstruction	26 (57.78)	3 (23.08)	>0.05
Rhinorrhea	31 (68.89)	5 (38.46)	>0.05
Sore throat	34 (75.56)	9 (69.23)	>0.05
Conjunctivitis	1 (2.22)	0 (0.00)	>0.05 *
Myalgia	31 (68.89)	4 (30.77)	<0.05
Diarrhea	4 (8.89)	1 (7.69)	>0.05
Rash	0 (0.00)	3 (23.08)	<0.01 *
Headaches	0 (0.00)	3 (23.08)	<0.01 *
Respiratory sample tested			
Nasopharyngeal swab	281	76	
Oropharyngeal swab	298	77	
Sampling days (Mean ± standard deviation)	6.69 ± 1.28	5.92 ± 2.06	

* By Fisher’s exact tests.

## Data Availability

All sequences generated in this study have been submitted to the National Genomics Data Center (https://bigd.big.ac.cn/, accessed on 10 November 2023) with submission number C_AA051888.1~C_AA051945.1. The nextflow pipeline for phylogenetic analysis and R scripts for all figures are available at https://github.com/Jinglu1982/ncov_cohort, accessed on 3 December 2023.
